# Belatacept in Pancreas Transplantation: Promising Insights From a Cohort Series

**DOI:** 10.3389/ti.2024.12778

**Published:** 2024-04-16

**Authors:** Christophe Masset, Claire Garandeau, Simon Ville, Magali Giral, Aurélie Houzet, Julien Branchereau, Ismaël Chelghaf, Benoit Mesnard, Gilles Blancho, Jacques Dantal, Diego Cantarovich

**Affiliations:** ^1^ Institut de Transplantation-Urologie-Néphrologie (ITUN), Nantes University Hospital, Nantes, France; ^2^ Nantes Université, INSERM, Center for Research in Transplantation and Translational Immunology, Unité Mixte de Recherche (UMR), Nantes, France

**Keywords:** pancreas transplantation, belatacept conversion, calcineurin inhibitor toxicity, pancreas allograft function, rejection

Dear Editors,

Belatacept has proven its efficacy as maintenance therapy in kidney transplant recipients (KTR), allowing a reduction in calcineurin inhibitor (CNI) allograft injuries. Despite being of interest for pancreas transplant recipients due to the β-cell toxicity of the CNI, data on the subject are scarce and suggest a high risk of pancreas rejection when used *de novo* [1].

We report our experience with 8 pancreas transplant recipients converted to belatacept (5 mg/kg day 1, 15, 28, and then monthly) during their follow-up, because of pancreas dysfunction (i.e., hyperglycemia not requiring insulin, *n* = 2) or kidney dysfunction (*n* = 6). The median time to conversion was 31 months Table 1. Of note, no systematic pancreatic biopsies were performed before conversion to rule out rejection episode. Nevertheless, among the 6 patients treated because of kidney dysfunction, 4 underwent a kidney allograft biopsy before belatacept in order to assess the etiology of dysfunction and rule out rejection.

**TABLE 1:** Summary of pancreas transplant recipients who underwent belatacept conversion.

Patient	Sex	Age at transplant	Category	Indication for conversion	Immunosuppressive regiment before belatacept	Time from belatacept introduction (months)	Total duration of belatacept at last follow-up (months)	Associated immunosuppression	eGFR at conversion (mL/min)	βcell function at conversion (Igls criterion)	Oral antidiabetics	Insulin	eGFR at 1 year (mL/min)	βcell function at 1 year (Igls criterion)	Occurrence of rejection and/or DSA	Infectious complication
1	F	28	SPK	Pancreas	CsA + low MPA	80	3	Low CsA + low MPA	87	Good	DPP4 inh	None	NA	NA	None	None
2	M	36	SPK	Pancreas	CsA + low MPA + steroids	10	36	Low mTOR inh. + low MPA	51	Marginal/Good	Metformin + GLP1-a	None	77	Optimal	None	None
3	F	35	PTA	Kidney	Tac + low MPA	9	20	Low Tac + low MPA	44	Optimal	None	None	55	Optimal	None	None
4	F	34	SPK	Kidney	Tac + low MPA	20	22	Low Tac + low MPA	49	Optimal	None	None	105	Optimal	None	None
5	M	47	SPK	Kidney	Tac + low MPA	150	4	Low MPA + low steroids	5	Optimal	None	None	NA	NA	None	None
6	M	53	PTA	Kidney	Tac + Steroids	48	40	Low mTOR inh. + low MPA	26	Optimal	None	None	39	Optimal	None	None
7	F	27	SPK	Kidney	Tac + Aza + Steroids	43	28	Low Tac + low MPA	24	Optimal	None	None	25	Optimal	None	None
8	F	49	SPK	Kidney	Tac + low MPA	1	30	Low Tac + low MPA	5	Good	None	None	18	Optimal	None	None

SPK, simultaneous pancreas kidney; PTA, pancreas transplant alone; CsA, cyclosporine; MPA, mycophenolic acid; Tac, tacrolimus; Aza, azathioprine; DPP4 Inh, DPP4 inhibitors; GLP1-a, GLP1-agonists.

Two patients were converted to belatacept in order to preserve β-cell function (Patient 1 and Patient 2). For Patient 1, Belatacept was interrupted 3 months later due to the patient’s convenience (refusal of injections). Patient 2 had a marginal β-cell function 2 years after transplantation related to the donor’s characteristics, persisting despite a switch from tacrolimus to CsA and addition of oral antidiabetics (metformin + GLP agonists). At belatacept conversion, CsA was withdrawn and replaced with low dose mTOR inhibitors in addition to low dose Mycophenolate Acid (MPA, 360 mg twice daily). At 2 years’ follow-up, we observed a significant improvement in fasting glycemia in addition to improvement in the kidney allograft function. HbA1c level decreased from 7.7% to 6% 2 years after conversion to belatacept, without any other medication modifications (and notably no change in his oral antidiabetic drugs).

Among the six patients converted for nephroprotection, there were 2 Pancreas Transplant Alone (PTA) and 4 Simultaneous Pancreas Kidney (SPK). Two SPK patients were on dialysis when initiating belatacept, (Patient 5 and Patient 8). Belatacept was interrupted after a few months in Patient 5 due to a poor renal prognosis and massive glomerulosclerosis and fibrosis on kidney biopsy. Causes of kidney impairment in other patients were CNI toxicity added to previous diabetic nephropathy in the PTA patients (Patient 3 and Patient 6), thrombotic microangiopathy related to CNI (Patient 4), sequelae of kidney allograft rejection (Patient 7) and kidney infarction in the immediate post-transplantation period (Patient 8). Associated immunosuppression was low tacrolimus (trough level between 3 and 5 ng/mL) plus low MPA (360 mg twice daily) in 4/6 patients and low everolimus (trough level between 3 and 5 ng/mL) plus low MPA in one patient. No steroids were used except for one patient who received neither CNI nor mTOR inhibitors.

Apart from Patient 5 who presented severe chronic injuries, belatacept conversion improved kidney allograft function in all patients. Notably, interruption of dialysis was allowed for Patient 8 who presented a primary non function following SPK transplantation due to ischemic complication. One year after conversion, the average improvement of estimated glomerular function (eGFR) was 20 mL/min (median = +13 mL/min), Supplementary Figure S1. All HbA1c levels remained excellent with optimal β cell function after conversion.

Importantly, during the complete follow-up (at least 18-month), we did not observe any suspicion of pancreas and/or kidney rejection nor appearance of donor specific antibodies (DSA). In our institution, patients are usually followed-up monthly following conversion, and pancreas rejection is suspected when unexplained significant elevation in lipasemia associated with glycemic imbalance. DSA were monitored yearly. Additionally, no serious infections were observed (notably no CMV/BKV), despite the use of low tacrolimus/mTor inhibitor in addition to belatacept.

Impairment of kidney function is not unusual in pancreas transplantation and might require CNI reduction. Even though mTOR inhibitors have been validated in a clinical trial conducted by our group, their use is associated with a wide range of side effects often leading to treatment interruption [2]. Moreover, the association of belatacept with a low dose of mTOR inhibitors, allows a significant improvement in pancreatic function and HbA1c in one patient with pancreatic dysfunction. Similar observations were made in recipients of islets transplant [3] or in diabetic KTR [4].

Importantly, no rejection episodes were observed among our patients. Even if we assume that the low number prevents any definitive conclusion, late conversion to belatacept may carry a lower risk of rejection compared to the *de novo* strategy. Moreover, the associated immunosuppression (mostly low-dose tacrolimus), probably participated in the prevention of rejection. A recent series of at-risk kidney transplant recipients converted to belatacept reported an eGFR improvement despite continuation of low-dose CNI [5]. Finally, no serious infectious complications were observed in our patients, suggesting that our strategy was quite efficient and safe.

In conclusion, our series highlights the feasibility of belatacept in pancreas transplant recipients. Whilst a larger dataset is obviously required, belatacept does allow CNI reduction (and even withdrawal), thus leading to improvement in kidney and pancreatic allograft functions. Importantly, we did not observe any pancreas/kidney rejection nor infectious complications, providing promising insights regarding its use in pancreatic and potentially islets transplantation.

## Data Availability

The original contributions presented in the study are included in the article/Supplementary Material, further inquiries can be directed to the corresponding author.

## Abbreviations

SPK, simultaneous pancreas-kidney; PAK, pancreas after kidney; PTA, pancreas transplantation alone; CNI, calcineurin inhibitors; CsA, cyclosporin A; MPA, mycophenolate acid; eGFR, estimated glomerular filtration rate.
