# Supplementary material for: Belatacept in Pancreas Transplantation: Promising Insights From a Cohort Series
Source: Transpl Int. 2024 Apr 16;37:12778. doi: 10.3389/ti.2024.12778 (PMC11058835; doi:10.3389/ti.2024.12778)
Supplement: Supplementary file 1 [file DataSheet1.docx]

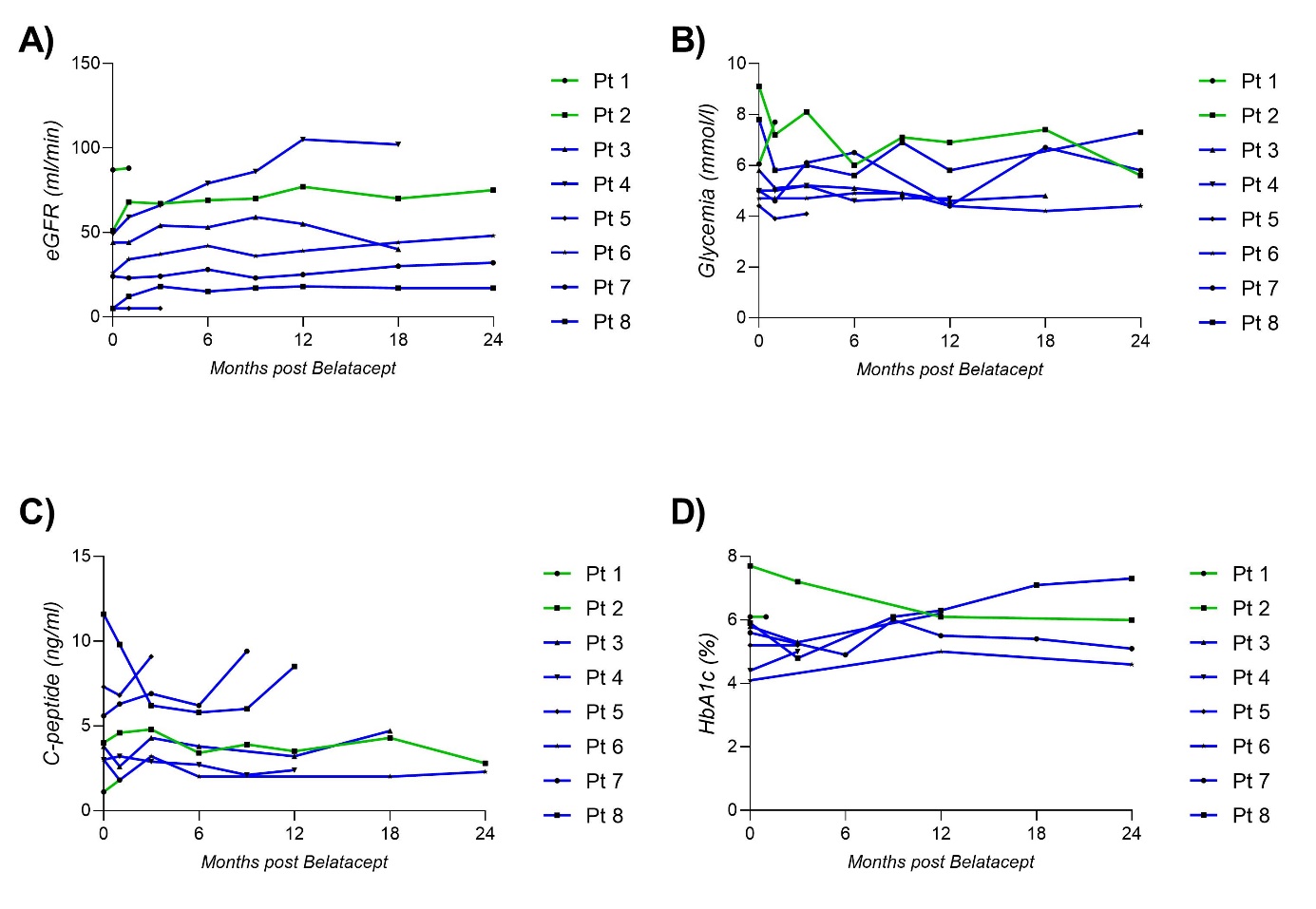


**Figure S1.** Evolution of eGFR (**A**), fasting glycemia (**B**), fasting C-peptide (**C**) and HbA1c (**D**) following introduction of belatacept in pancreas transplant recipients (green : pancreas indication, blue : kidney indication).


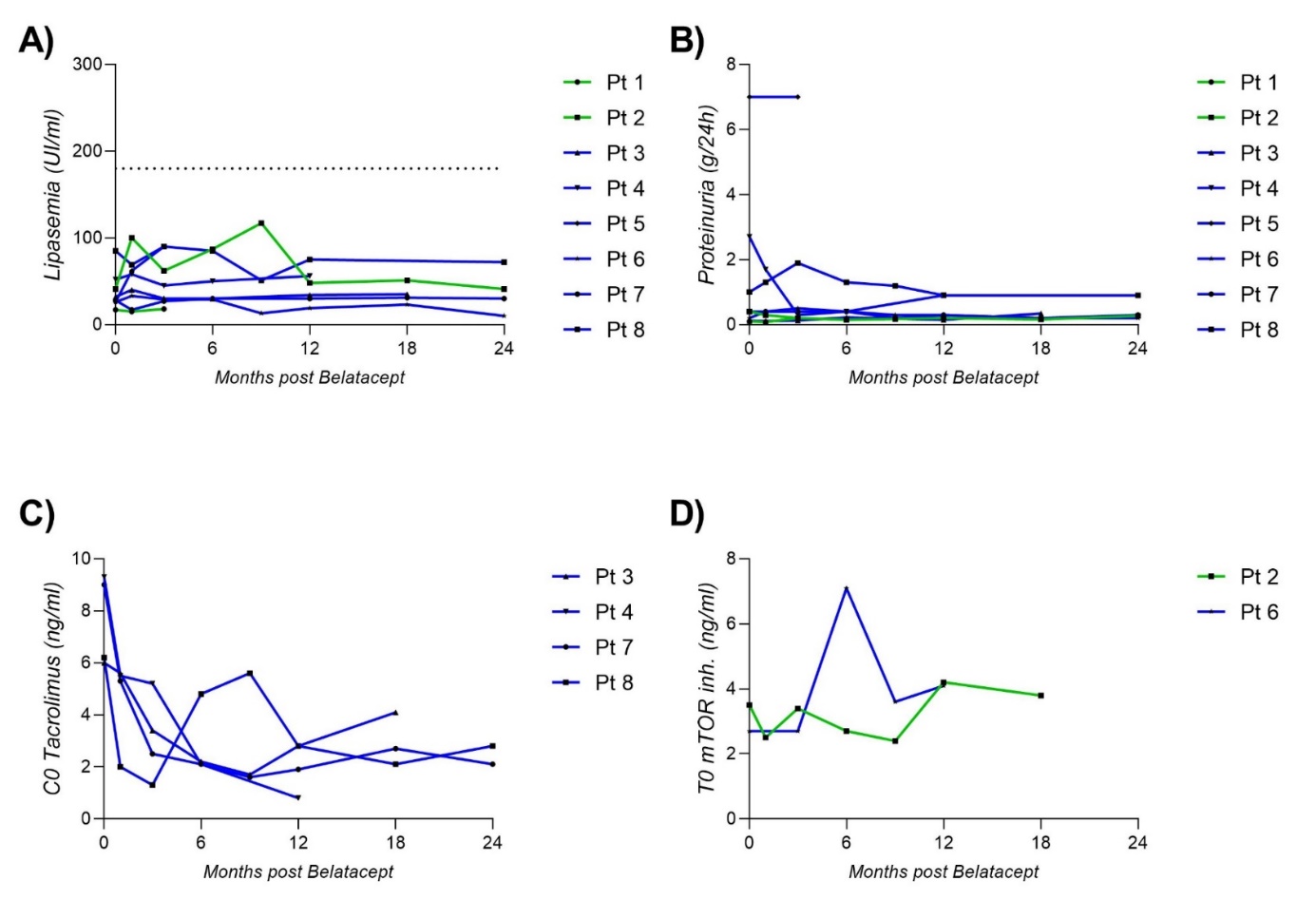


**Figure S2**. Evolution of lipasemia – the dotted line represents the threshold of 3 times normal values(**A**), proteinuria (**B**), tacrolimus trough levels (**C**) and mTOR inhibitors trough levels(**D**) following introduction of belatacept in pancreas transplant recipients (green : pancreas indication, blue : kidney indication).
